# Evolutionary context of psoriatic immune skin response

**DOI:** 10.1093/emph/eoab042

**Published:** 2021-12-01

**Authors:** Izzy Starr, Kristina Seiffert-Sinha, Animesh A Sinha, Omer Gokcumen

**Affiliations:** 1 Department of Biological Sciences, University at Buffalo, The State University of New York, Buffalo, NY, USA; 2 Department of Dermatology, University at Buffalo, The State University of New York, Buffalo, NY, USA

**Keywords:** psoriasis, evolutionary medicine, skin homeostasis, anthropology

## Abstract

The skin is vital for protecting the body and perceiving external stimuli in the environment. Ability to adapt between environments is in part based on skin phenotypic plasticity, indicating evolved homeostasis between skin and environment. This homeostasis reflects the greater relationship between the body and the environment, and disruptions in this balance may lead to accumulation of susceptibility factors for autoimmune conditions like psoriasis. In this study, we examined the relationship between rapid, lineage-specific evolution of human skin and formation of psoriatic skin responses at the transcriptome level. We collected skin tissue biopsies from individuals with psoriasis and compared gene expression in psoriatic plaques to non-plaque psoriatic skin. We then compared these data with non-psoriatic skin transcriptome data from multiple primate species. We found 67 genes showing human-specific skin expression that are also differentially regulated in psoriatic skin; these genes are significantly enriched for skin barrier function, immunity and neuronal development. We identified six gene clusters with differential expression in the context of human evolution and psoriasis, suggesting underlying regulatory mechanisms in these loci. Human and psoriasis-specific enrichment of neuroimmune genes shows the importance of the ongoing evolved homeostatic relationship between skin and external environment. These results have implications for both evolutionary medicine and public health, using transcriptomic data to acknowledge the importance of an individual’s surroundings on their overall health.

## Lay Summary

The skin is important for protecting the body from the environment and perceiving external stimuli, creating an evolved balance between skin and the environment. We compare skin gene expression in humans with psoriasis to humans and non-human primates without psoriasis to better understand human-specific evolutionary changes in the skin. Our results suggest important evolutionary links between skin perception, human-specific skin development and immune response.

## BACKGROUND AND OBJECTIVES

Skin is the largest organ and one of the primary ways we interact with the world. Skin is simultaneously a barrier protecting the rest of the body from external factors and a primary sense organ allowing those external factors to be perceived and experienced. Skin is composed of various cell types creating chemical, physical, and immune barriers moderated by cutaneous nerve interactions [1, 2]. The skin microbiome is an additional host-specific immune barrier that has co-evolved with skin [3, 4]. The skin is under high evolutionary pressure simultaneously acting to increase skin immune capability, barrier function and sensation, while maintaining the adaptive and dynamic homeostatic relationships between the skin, skin microbiome and external environment [1, 5–7].

The evolution of a dynamic homeostasis between the skin and the environment allows for movement between and adaptation to different ecologies within individual, community and species lifetimes. Technologies like fire, clothing, agriculture and medicine have impacted human survival in a vast range of ecologies and play crucial roles in ongoing human relationships with the environment, likely coinciding with probably the single most important biological change in the skin physiology: hair loss [8, 9]. This likely impacted the evolution of multiple skin-level phenotypic differences between humans and non-human primates, including increased skin thickness and oiliness, as well as differential skin microbiome composition [3, 6, 10, 11]. The balance between human skin and the environment has not evolved in a vacuum. This continuous process depends on many simultaneously varying parameters, including constantly changing environments and models of cultural and technological buffering. Thus, the evolution of the homeostatic balance between the skin and environment is a non-deterministic and ever-changing process. Based on these points, we hypothesize that human skin’s role as a dynamic protective and perceptive barrier is manifested in human-specific genetic changes in the skin when compared to non-human relatives, and that human-specific gene regulation of this barrier organ can give rise to complex human-specific autoimmune skin conditions like psoriasis. To test this hypothesis, we examine human skin using two levels of change: evolutionary differences in human and non-human primate skin and individual-level differences between lesional and non-lesional skin in people diagnosed with psoriasis. By linking these interwoven timescales, we find biomedical implications for the evolution of environmental skin perceptivity within the context of psoriasis.

Psoriasis is a genetically and environmentally linked immune-mediated skin condition with a variety of clinical manifestations. The most common subtype of psoriasis, plaque psoriasis, is characterized by general inflammation and buildup of well-demarcated, erythematous (reddish) plaques with overlying, coarse scales in the skin. Psoriasis is found at rates of ∼2–4% across all human populations, and is more common in adults than children; the global prevalence of psoriasis is increasing [12, 13]. Several regions across the human genome have been identified for their roles in psoriasis susceptibility, as well as skin barrier and immune function [14, 15]. Psoriasis severity, symptom development and healing have been correlated with environmental risk factors, including infections, medications, toxin exposure and negative social interactions [12, 13, 16, 17]. Biblical influence on the historical record has linked psoriasis and other chronic skin conditions with leprosy, a contagious mycobacterial infection; underlying attitudes surrounding this identify skin conditions as ‘afflictions’ reflecting personal moral failure [18]. This has a current impact on how individuals with visible skin differences are treated by others, impacting psoriasis development and severity, which are often late-onset and exacerbated by chronic stress [19–21].

While ‘psoriasis’ is currently considered a skin-specific autoimmune issue, increasing evidence supports the recognition of psoriasis as a multisystem chronic inflammatory condition. This is reflected by multiple associated comorbidities, including autoimmune arthritis, metabolic syndromes, cardiovascular disease and mental illness, where chronic inflammation and stress can lead to increased susceptibility to external stimuli and altered immune function [13]. The chronic immune response associated with psoriasis can be present even when the skin appears ‘normal’ or ‘asymptomatic’ (Research in sociological and biomedical fields finds that descriptors like ‘normal’ and ‘healthy’ create an implicit moral binary toward a certain appearance or experience, adversely impacting individuals who do not meet this standard (as discussed by Metz and Kirkland ‘Against Health: How Health Became the New Morality’ (2010, NYU Press). In skin, these descriptors do not accurately describe observations about the skin and are subjective from clinician to clinician. As such, this article uses ‘lesional’ or ‘plaque’ and ‘nonlesional’ or ‘non-plaque’ to describe observable states of psoriatic immune response in the skin.). Given that psoriasis plays a role in the skin-environment perception mechanism, we are curious about human-specific evolutionary changes in skin that may relate to the development of chronic and systemic skin immune responses.

In this article, we compare skin transcriptome data from non-human primates and humans with and without psoriasis to identify genes involved in human-specific skin evolution. We seek to contextualize an increasingly common human experience from multiple evolutionary angles, given that complex systemic autoimmune conditions can neither be simplified nor separated from lived experience or evolutionary history. We find that genes enriched in human-specific skin evolution also have implications in psoriatic immunity, likely exacerbated by stressful individual and global environments. It is our hope that by recognizing evolutionary and biomedical connections between the past and the present, this article will provide a framework for broader studies that integrate human evolution, skin development and immune susceptibility and response.

## METHODOLOGY

### Sample collection

Three patients (Supplementary Table S1) with a confirmed diagnosis of mild-moderate plaque psoriasis based on clinical examination and histological features were enrolled into an IRB-approved study on genetic, microbial and immune response variation in psoriasis (MODCR00005370). To determine the severity of each participant’s psoriasis, we used the field-standard PASI [22], which produces a numeric score ranging from 0 to 72 based on the severity of the lesion and plaque characteristics, as well as the size of the affected skin area. We provide the PASI scores for individual samples in Supplementary Table S1.

After signing informed consent, the patients underwent a detailed skin examination and provided demographic and epidemiologic information. Full-thickness skin samples were collected by punch biopsy from lesional and site-matched non-lesional skin. Biopsies were stored in RNAlater storage solution.

### RNA sequencing and analysis

Samples (Supplementary Table S2) were processed by GENEWIZ. RNA sequencing was performed via Illumina HiSeq, 2 ×150 bp configuration. Quality control of paired reads was performed using FastQC and MultiQC [23, 24]. Illumina adaptor sequences and short or low quality sequences were further filtered using Trim Galore! [25]. Reads were aligned to the human reference genome (hg38) using Kallisto [26]. Aligned data were then comparatively analyzed using the DESeq2 pipeline [Benjamini and Hochberg false discovery rate (FDR) corrected adjusted *P*-value, *P* < 0.05], where psoriatic plaques were compared to non-psoriatic tissue as a control without inputting information of the sample of origin [27]. In parallel, we conducted a similar comparison, this time incorporating the paired sample information and found 2506 differentially expressed genes. The set of significantly differentially expressed genes detected by the more conservative former approach was essentially encompassed by that detected by the latter approach (1304/1396, 93%) genes. Even though we provide the results from both approaches (Supplementary Table S3), for the follow-up analysis, we used the more conservative non-paired approach.

We compared our list of differentially expressed genes in psoriatic lesional skin with previous studies to ensure that our analysis is concordant with previously published RNAseq datasets. We found that 55 out of 100 genes with the most significant expression differences between lesional and non-lesional skin in our dataset were also highlighted in the top 100 most significant hits in three other comparable studies (Supplementary Fig. S1A and B) [28–30]. This is a considerable overlap given the well-known technical, biological and statistical variation in transcriptomics studies [31, 32]. For example, prior RNAseq studies involving psoriasis differ from each other and this study in the number of samples that they used, the statistical methods used to identify differentially expressed genes, and the thresholds for differential expression. In fact, two of these studies used Wilcoxon rank-sum tests to identify differential expression without invoking more sophisticated methods to account for inherent noise in RNAseq studies [29, 30]. The DESeq2 framework that we apply here is state-of-the-art in comparative RNAseq analysis and we argue that our dataset provides a precise look at the expression changes in lesional skin.

### Arakawa primate analysis

The primate skin transcriptome data were downloaded from the DDBJ Sequence Read Archives (accessions DRX121122–DRX121135) [33]. These data include skin tissue biopsies from five humans without psoriasis, three chimpanzees, two gorillas and three orangutans (Supplementary Table S2). We aligned the raw RNAseq data from each species to the human reference genome (hg38) so that orthologous genes could be readily compared. In parallel, the same data were aligned to the respective primate reference genomes (PanTro3, gorGor4 and Ppyg2) to check for systematic biases that could be introduced via misalignment and/or reference genome quality [34]. These alignment approaches yielded similar results (Supplementary Fig. S2). The human reference genome-aligned dataset was used for downstream analysis.

### Data analysis

Data analysis and visualization was conducted using Rstudio v1.3 with R v4.0.3 using the following packages: tidyverse (v1.3), DESeq2 (v1.12.3), tximport (v1.24.0), biomaRt (v2.40.5), ggplot2 (v3.3.2), ggpubr (v0.4.0), RColorBrewer (v1.1-2), circlize (v0.4.12) and waffle (v.0.7.0).

Functional enrichment categories were determined using ShinyGo (FDR < 0.05, top 20 genes) [35]. Figures were curated using BioRender.com.

Gene clusters were examined by mapping reads to the human reference genome (hg38) using bowtie2 (v2.4.4) and samtools (v1.10). We then analyzed the read-depths and paralogous gene alignments of the respective samples using IGV (v2.11.0).

### Identifying clusters of differential gene expression

We defined a genomic region as a cluster of differential expression if, in that locus, we identified two or more genes that are differentially expressed between human and chimpanzee skin, and two or more genes that are differentially expressed between psoriatic plaques and non-lesional skin. We identified six such clusters, all of which harbor closely related gene families. The members of these gene families may be close enough in sequence content that short RNAseq reads may not uniquely map to individual genes. We chose our mapping and quantification approach specifically to account for such ambiguous reads. Specifically, Kallisto uses an ‘Expectation Maximization’ method to deal with multi-mapped reads. This approach recognizes uniquely mapped reads to differentiate between genes with otherwise similar sequence content. Similarly, DESeq2 uses a sophisticated comparative framework where the read-depths are internally normalized and multiple-hypothesis aware significance is calculated across all samples. To further investigate whether this issue may cause bias in our analysis, we manually checked the presence of segmental duplications and gene annotations both within the human genome and also between human and chimpanzee reference genomes for the clusters that we highlight in the manuscript (Supplementary Fig. S3). Based on this analysis, we found three segmental duplications, indicating recent homologous duplications encompassing *LCE1E* and *LCE1D*; *S100A7* and *S100A7A*; *SERPINB4* and *SERPINB3*; *KRT81* and *KRT86*; *KRT6A*, *KRT6B* and *KRT6C*.

We manually checked the alignments of the RNAseq reads from psoriatic lesional and non-lesional samples as well as non-psoriatic human and chimpanzee samples to the human reference genome (hg38) using IGV (v2.11.0). We observed that in each case, the mapping is unambiguous and each gene can be differentiated from each other. We provide a screenshot of the *SERPINB4* and *SERPINB3*, which are relatively recent duplicates of each other, as an example (Supplementary Fig. S4). In our cross-species analysis, we found only one lineage-specific whole gene duplication, *Loc112206374*, which seems to be a chimpanzee specific duplication of *KLK5* gene based on sequence similarity (Supplementary Fig. S3). We found no significant expression differences of *KLK5* between human and chimpanzee, or psoriatic and non-psoriatic tissues. In addition to duplications of whole genes, partial duplications affecting genes may also lead to potential biases. For example, epidermal differentiation complex (EDC) genes *FLG* and *FLG2* (among others), as well as protocadherin exons, harbor multiple large exonic repeats that are more than 90% identical to each other. Even though we believe that our results are robust, we believe that it would be pertinent to design future studies to use longer sequence reads to more accurately capture the RNA abundances in these more complicated loci.

## RESULTS

### Genes expression differences in psoriasis are implicated in recent human evolution

**Figure 1.**
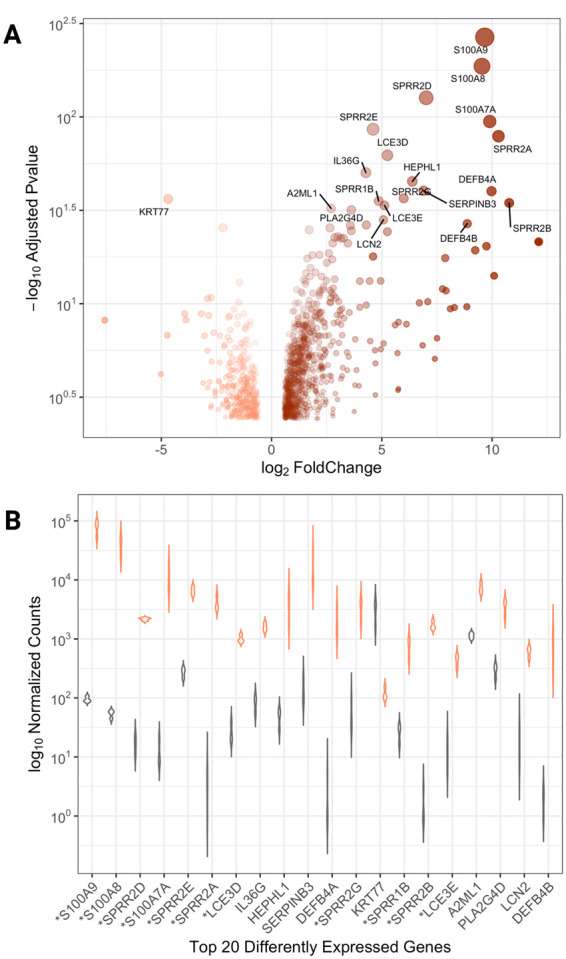
Comparison of differentially expressed genes in psoriasis (**A**) A volcano plot depicting the significance of genes that are upregulated (brown) and downregulated (salmon) in psoriatic plaque and non-plaque skin. (**B**) A violin chart comparing normalized gene counts in the top 20 differentially expressed genes in psoriatic lesional (salmon) and non-lesional (grey) skin tissues. EDC genes are identified with asterisks

In this study, we aim to develop a framework for understanding changes in psoriatic immune response and gene expression in the context of primate evolution. First, we used novel skin transcriptome data from individuals with psoriasis and identified genes with significant expression differences between skin with and without psoriatic plaques (948 upregulated and 402 downregulated in psoriatic plaques) (Fig. 1A and Supplementary Table S3). These results are consistent with previous RNAseq studies (Methodology and Supplementary Fig. S1A and B) and captures the well-characterized transcriptome markers for psoriasis, such as members of the EDC (e.g. *S100A12*, *S100A7A* and *LCE3E*), defensin (e.g. *DEFB4A*), *SERPIN* (e.g. *SERPINB4*) gene families [28–30, 36]. We conducted a functional enrichment analysis using a FDR < 0.05 (Supplementary Table S4). We observed that upregulated genes are significantly enriched in processes related to the immune system, mineral and ion metabolism and generalized responses to external and internal stimuli; downregulated genes are enriched in cellular and biological adhesion, morphogenesis and other developmental processes. Reflecting previous research on the psoriatic transcriptome, our data tilt toward broad upregulation of genes involved in immune response, skin cell proliferation and differentiation and skin barrier function [28–30]. The enrichment of these functional categories fits with a model where psoriatic immune response is a result of genetically predisposed skin cells responding to increased barrier and immune stress. The skin in a psoriasis-stressed state responds with increased thickening, general inflammation and increased skin microbiome fluctuation [37, 38].

Genes with the most dramatic regulatory expression changes in psoriatic plaques are from protein-coding gene families in the EDC, a 2 Mb gene cluster on Chromosome 1. The EDC codes for proteins that are crucial in the construction of the skin barrier as a complex structural, protective, sensory and ecological composite organ [39]. Evolutionary pressures acting on this region affect point mutations, structural variants and regulatory elements strongly associated with skin barrier function and skin immune response in mammalian, primate and human evolutionary trajectories [40]. The EDC has been recognized as a psoriasis susceptibility locus (PSOR4), where genetic variants within the EDC have been maintained in the human lineage via a balancing effect between increased immune response and decreased wound-healing ability [6, 41–43]. We find that 23 out of 60 genes from several EDC gene families are differentially expressed in lesional versus non-lesional skin, showing upwards of a 1000-fold increase in psoriatic plaques (Fig. 1B). Consistent with previous studies linking EDC genes to psoriasis, differentially expressed genes in this locus include members of the *S100* gene family (calcium-binding and regulation of cellular function) [44], late cornified envelope (*LCE*) gene family (broad-spectrum antimicrobial activity) [41, 43, 45], and small proline-rich (*SPRR*) gene family (cell cornified envelope structural function) [46]. Given that multiple genes and gene families within the EDC locus have similar patterns of regulation in human skin and other epithelial tissues, it is possible that the network of regulatory elements in the EDC is mirrored in other regions of the genome with relevance to skin and autoimmune evolution, as recently suggested [40, 47].

Overall, our dataset replicates previous work to a large extent (Supplementary Fig. S1B), capturing key gene families and specific transcriptomic markers. Therefore, it provides a robust empirical framework to delineate the relationship between psoriatic expression in human skin in comparison to other primate species. Particularly, we note that the EDC locus is a hotspot for differential expression when lesional and non-lesional skin are compared and that the genes in the same locus have been shown to be evolving under selective forces in the human lineage, including *FLG*, *LCE3B, LCE3C, TCHHL1*, *SPRR4*, *LELP1* and *S100A2* [6, 48, 49]. Based on these observations, we hypothesize that the same loci that are involved in psoriatic skin immune response may overlap with loci that are evolving in a lineage-specific manner in humans. To test this hypothesis, we wanted to document differences in skin gene expression trends between extant non-human primate species and more specifically identify human-specific gene expression trends impacting skin immunity and psoriasis.

### Differentially regulated genes in human skin

To bolster our framework, we use the same analytic pipeline to identify changes in gene expression in skin from humans and non-human primates without psoriasis by mapping and quantifying RNA sequencing data from Arakawa *et al.* (see Methodology) [33]. We used a different mapping and quantifying approach to analyze both our psoriasis dataset and the Arakawa dataset to control for any possible introduction of bias due to differences in sample collection and processing. We mapped all reads to the human reference genome (hg38) and quantified their abundances using Kallisto, followed by comparative analysis using DESeq2. We identified genes with significant (*P*_adj_ < 0.05) expression differences between human skin and chimpanzee skin, finding 686 downregulated genes and 816 upregulated genes, respectively (Supplementary Table S5). Genes that are significantly downregulated in non-psoriatic human skin compared to chimpanzee skin are enriched in keratinization, and skin cell differentiation and development functional categories (Supplementary Table S4). This downregulation may reflect gene expression changes associated with phenotypic skin differences between humans and non-human primates, including skin thickness, hairiness and oiliness [10, 50]. This cross-species empirical framework allows further understanding of how human-specific functional changes in gene expression may be related to immune-mediated skin conditions.

### The intersection of evolutionary and epidemiological expression trends in human skin highlight immune response and neurological pathways

**Figure 2.**
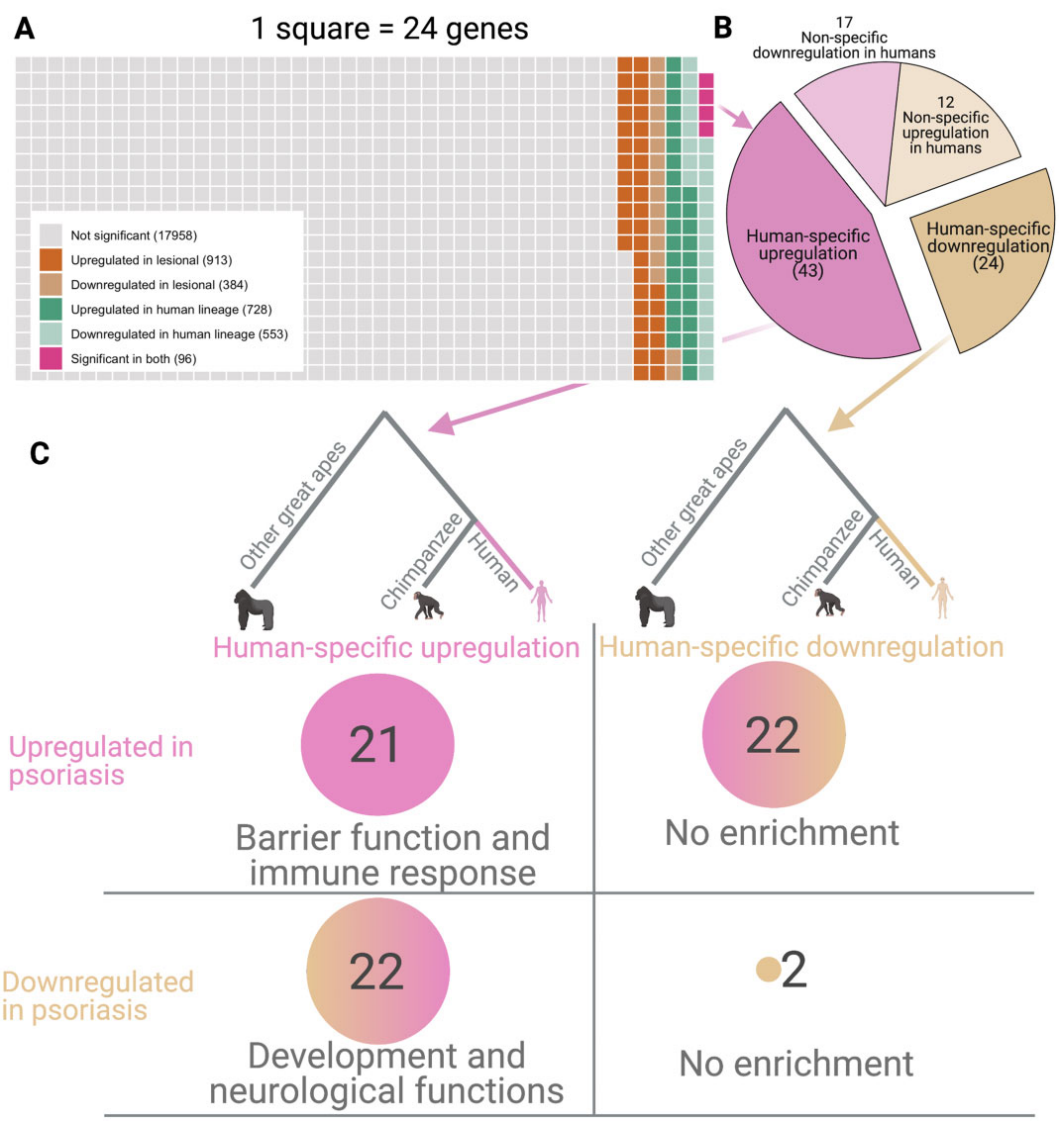
Comparison of differentially expressed genes in psoriasis and human lineage (**A**) A waffle plot depicting the proportion of differentially expressed genes in psoriatic plaques compared to non-lesional skin (shades of brown) and in non-psoriatic human skin compared to chimpanzee skin (shades of green). Lavender boxes indicate genes that are differentially expressed in both comparisons. (**B**) A pie chart indicating the proportion of genes that are significantly upregulated or downregulated in humans as compared to chimpanzees. The genes where the change in expression is specific to chimpanzees or variable among great apes are dubbed non-specific and shown in lighter colored pie sections. (**C**) Breakdown of genes showing human-specific and psoriasis-specific differential regulation; upregulated genes are indicated in pink and downregulated genes are in tan

We identified differentially expressed genes in human skin without psoriasis compared to chimpanzee skin that also show expression differences in psoriatic lesional skin compared to non-lesional skin, and found 96 genes fitting these criteria (Fig. 2A). We then used gene expression data from gorilla and orangutan skin to filter this dataset, identifying 67 genes that show significantly higher or lower expression in human skin as compared to other great apes (Fig. 2B and Supplementary Table S6, see Methodology). We found 43 genes upregulated and 24 genes downregulated in human skin in a species-specific manner (Fig. 2C).

We further categorized these genes based on their expression trends in psoriatic skin. We found that the 21 genes that are upregulated in the human lineage and further upregulated in psoriatic plaques are strongly enriched for functional categories involved in response to stress (Fig. 2C and Supplementary Table S4). Among these, we note *LTF*, *DNASE1L3*, *NFASC*, *TRIM16* and *NME2*, which involve neutrophil activation and/or keratinocyte differentiation, and are associated with systemic immune-mediated conditions [51, 52]. For example, *NME2* is involved in both of these processes through CD4 activation and skin repair and is expressed ∼15-fold higher in non-psoriatic human skin and ∼1.7-fold higher in psoriatic plaques [13]. One possible explanation for this expression trend is that human skin is both more and less exposed to the environment due to fur loss and clothing use than other great apes, which may lead to adaptive evolution of higher expression in genes, such as *NME2* that activate a stronger response to external stimuli [53].

In contrast, and to our surprise, the 22 genes upregulated in the human lineage but downregulated in psoriatic skin are enriched for sensory system development (Supplementary Table S4). These genes include *SOX8*, *MYOM1*, *BCAR3* and *PTPRM*, which are linked to cellular adhesion and neuronal development and function in multiple tissues [54–57]. For example, *PTPRM* is upregulated ∼5.6-fold in non-psoriatic human skin but is downregulated ∼1.7-fold in psoriatic plaques. *PTPRM* is a well-studied tyrosine phosphatase enzyme implicated in regulating axonal guidance and cellular adhesion; it may have complex associations with other systemic autoimmune conditions, including lupus [54, 58]. It is possible that at the intersection of cell-adhesion, neural-cell migration and immune-system involvement, the regulation of the expression of these genes may underlie the reported greater skin sensitivity and the feedback loop that further proliferates local immune response [5, 59, 60].

### Overlap between psoriasis and human-specific skin regulation

**Figure 3.**
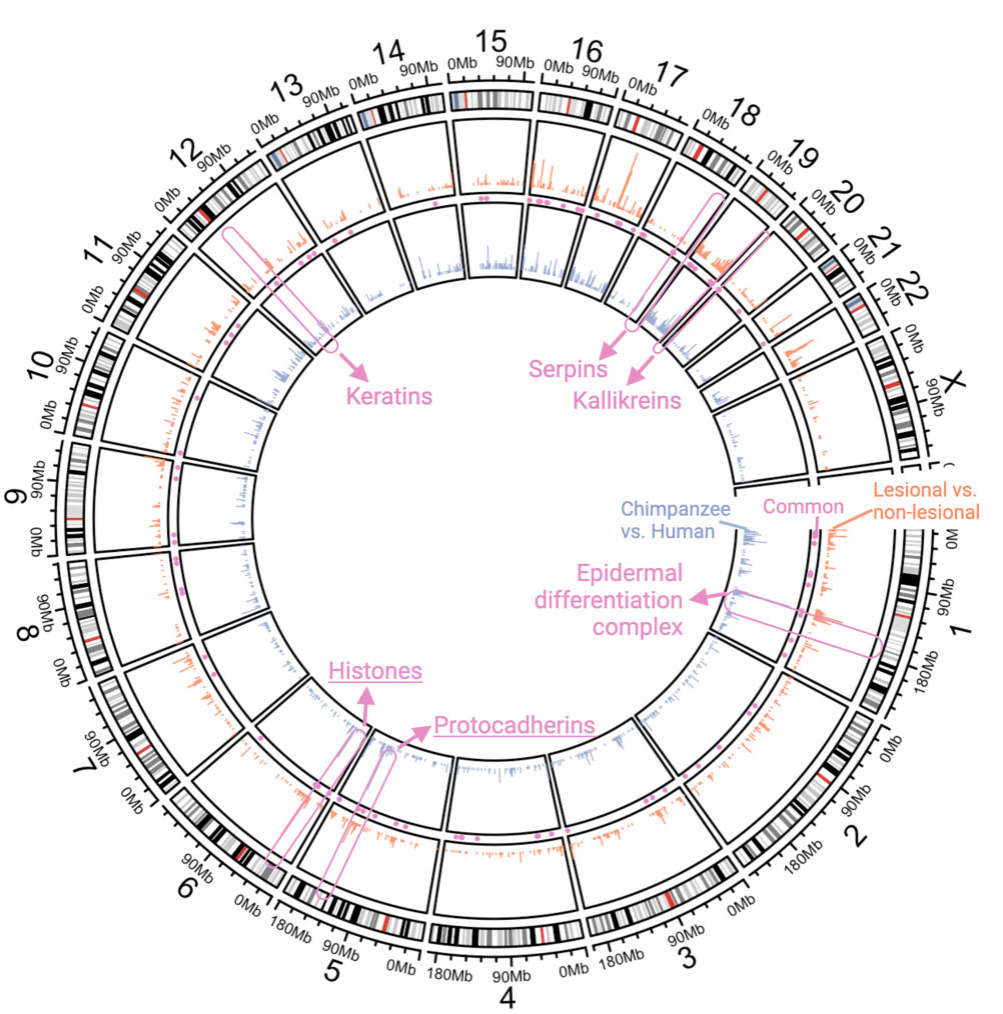
Genomic clusters of genes involved in psoriasis and human skin evolution A circos plot showing the density of all expressed genes. The outer ring shows cartoon chromosomal karyotypes and chromosomal locations. The next circle toward the center shows histograms (salmon) of differentially expressed genes (down- and upregulated in psoriatic plaques compared to non-lesional skin). The next circle includes histograms (blue) of differentially expressed genes between non-psoriatic human and chimpanzee skin. Clusters where multiple genes are differentially expressed in both human lineage and psoriasis contexts are indicated in the center (pink)

Next, we hypothesized that human-specific and psoriasis-specific changes in gene expression may be driven by the same or similar regulatory architecture to the EDC. If correct, we expect to observe genomic regions where genes with human-specific and psoriasis-specific expression co-localize. Indeed, we observed six gene clusters (see Methodology) across the human genome where multiple genes are differentially expressed in non-psoriatic human skin compared to chimpanzee skin and in psoriatic plaques when compared to non-lesional skin from individuals with psoriasis. These gene clusters include the EDC (Chromosome 1), a protocadherin gene cluster (Chromosome 5), a histone gene cluster (Chromosome 6), a keratin gene cluster (Chromosome 12), a serpin gene cluster (Chromosome 18) and a kallikrein gene cluster (Chromosome 19) (Fig. 3).

**Figure 4.**
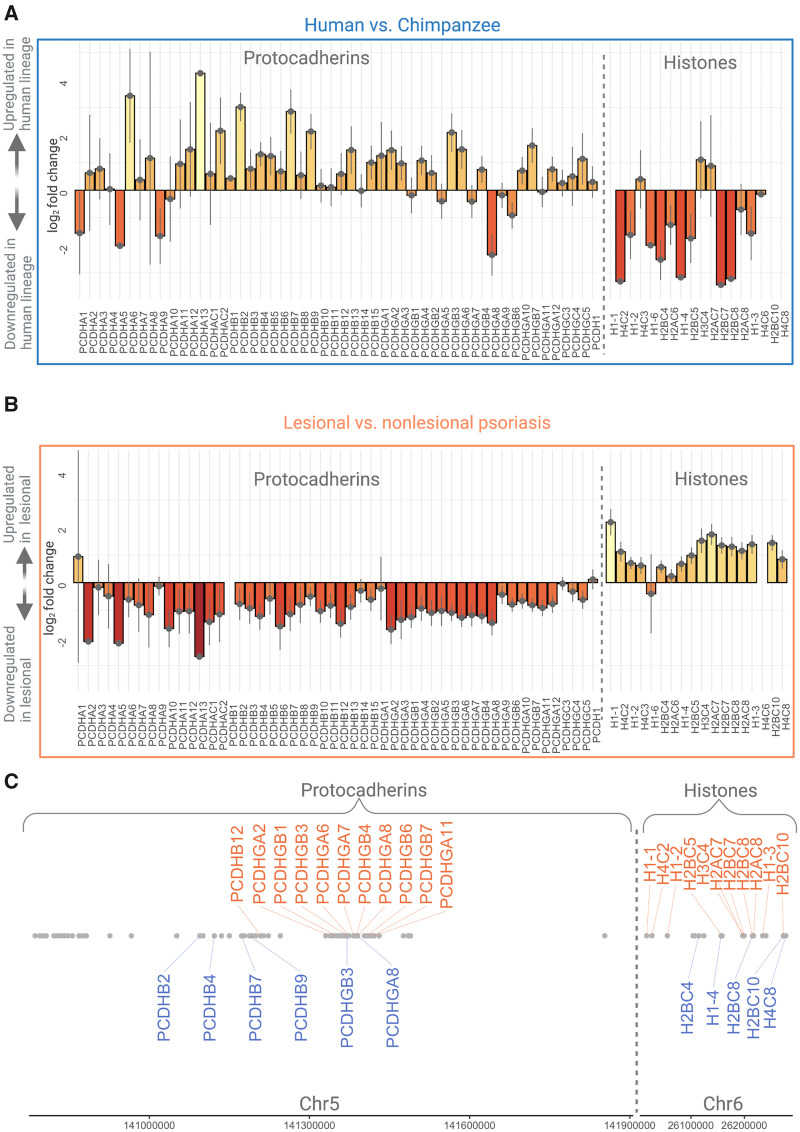
Gene expression in protocadherin and histone clusters The barplots show the log2 fold changes of gene expression in the human lineage (**A**) and in psoriasis (**B**). We highlight two clusters, a protocadherin cluster on Chromosome 5 and a histone cluster on Chromosome 6, which exemplify cases where the regulatory change is unidirectional, albeit opposite in human evolution and psoriasis. (**C**) The chromosomal location of genes, where those with significant human-lineage and psoriatic-expression differences match panels (A) and (B)

To explain these clusters, we first considered an overarching regulatory mechanism that triggers multiple genes in the same cluster to be up- or downregulated in the human lineage and/or during psoriasis. In this case, we expect that genes in a given cluster will be consistently expressed in the same direction for a given human-specific or psoriasis-specific state. A cluster of 18 histone genes on a 269 kb segment on human Chromosome 6 fits this expectation (Fig. 4C). We found that 13 (72%) histone genes are downregulated (5 of them significantly, *P*_adj_ < 0.05) in human skin (Fig. 4A), and 16 (∼89%) of the genes in this cluster are upregulated (15 of them significantly, *P*_adj_ < 0.05) in psoriatic plaques (Fig. 4B). For example, Arakawa *et al.* suggest that transcription factors may impact histone modification in skin at a histone protein level, so it is interesting to observe changes in histone expression at a gene level as well, especially in comparison with an immune skin condition that is not found in non-human primates [33]. There is an emerging body of work suggesting that the abundance of specific histones, which are highly conserved proteins involved in the process of chromatin packaging, may affect tissue-specific gene expression changes, including immune response [61–64]. It is plausible that the expression trends of histone genes may have evolutionary and biological effects, particularly in relationship to immune response in so-called ‘disease states’. The exact mechanisms through which expression differences of histones affect phenotypic traits, human skin characteristics and psoriasis biology remain exciting future venues of research.

A family of 52 protocadherin transcripts on human Chromosome 5 also exhibit co-localization of human-specific and psoriasis-specific gene expression (Fig. 4C). This cluster shows general upregulation in the human lineage (Fig. 4A), but revert back to lower levels of expression in psoriatic lesions (Fig. 4B); of the 52-gene protocadherin cluster, 40 (77%) are upregulated (5 of them significantly, *P*_adj_ < 0.05) in non-psoriatic human skin, and 49 (94%) are downregulated (11 of them significantly, *P*_adj_ < 0.05) in psoriatic plaques. Protocadherins are primarily expressed in the nervous system; the large protocadherin gene family is associated with an immunoglobulin-like ability to combine different transcripts to create single-cell identity of neurons [61, 62]. The highly repetitive nature of this region along with a high level of splicing variation make delineating the exact functional effects difficult using only short RNAseq sequences. Thus, future studies employing long-read sequencing platforms at the single-cell level provide an exciting venue to understand the role of neuroimmune pathways in skin evolution and psoriasis.

### EDC and keratin clusters are central to both skin evolution and psoriasis plaque formation through regulation of different genes

**Figure 5.**
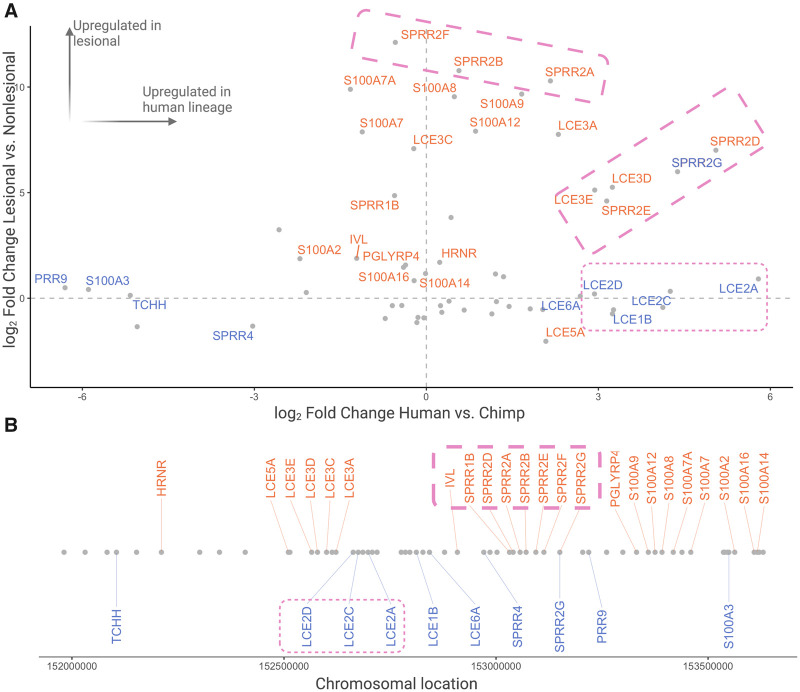
Expression changes in EDC genes in psoriasis and in human evolution (**A**) A scatterplot showing gene expression differences observed between non-psoriatic human and chimpanzee skin on the *x*-axis, and plaque and non-plaque psoriatic skin on the *y*-axis. Genes are identified by significant (*P*_adj_ < 0.05) differences in the human-lineage (blue) and psoriatic lesions (salmon). Two clusters with similar expression trends are marked by dotted rectangles. (**B**) The chromosomal location of EDC genes, where genes with significant human-lineage and psoriatic-expression differences match panel (A). Two EDC subclusters are indicated by dotted pink rectangles

The EDC, as we discussed earlier in this manuscript, as well as the keratin gene family, may be the most important regions in the genome for both human skin barrier evolution and psoriasis [6, 40, 41, 43, 45, 63]. Our results confirm the dramatic psoriasis-specific upregulation of multiple genes implicated in wound healing and stress response: LCE genes, calcium-binding S100 genes and proline-rich SPRR genes (Fig. 5A) [46, 64]. We initially hypothesized that some of this upregulation is facilitated by regulatory evolution in the human lineage as a part of fur loss and consequent thickening of the epidermal barrier. If so, we would expect the same genes that are upregulated in the human lineage to be further upregulated in psoriatic plaques.

Instead of consistent patterns of regulation across the EDC region, we found specific subclusters of physically adjacent genes within the EDC showing similar expression trends (Fig. 5B). Certain cornified envelope genes, *LCE2A*, *LCE2C* and *LCE2D*, are all upregulated in the human lineage but show no change in psoriatic plaques. In contrast, other cornified envelope genes, *SPRR2G*, *SPRR2D*, *SPRR2E*, *LCE3D* and *LCE3E*, are upregulated both in the human lineage and in psoriatic plaques. We found one gene, *SPRR2G* to be significantly upregulated in psoriatic plaques as compared to non-lesional psoriatic skin and in non-psoriatic human skin as compared to chimpanzee skin. It is plausible that localized regulatory elements further fine-tune specific expression trends in this locus in primate skin [47]. The complex interaction of EDC genes and immune response in the context of psoriasis has been well studied and it is clear that this locus is a central hub of molecular innovation in human skin barrier evolution [6, 40, 41, 43, 63]. However, the exact ways in which each gene contributes to the characteristics of human skin and how recent evolution affects psoriatic plaque formation remain fascinating future avenues of research.

Keratins are filament-forming proteins involved in the construction of the outer layer of the skin, filamentous growths like hair and nails, and developmental and immune processes [65]. There are more than 50 keratin genes in multiple clusters across the human genome, likely evolved through independent gene duplication events. We identify one such cluster on Chromosome 12 harboring 27 protein-coding genes (Supplementary Fig. S5C), where 11 of these genes are downregulated in human skin (Supplementary Fig. S5A) and 3 genes show differential expression in psoriatic plaques (*P*_adj_ < 0.05) (Supplementary Fig. S5B). Our results suggest that evolved human-specific regulatory changes do not necessarily overlap with the regulatory changes occurring in psoriatic plaque formation in this particular keratin cluster.

Closer inspection further suggests that the downregulated keratin genes in non-psoriatic human skin (keratins 71–75 and keratins 81–86) are involved in ‘hard’ keratinization and cornification, likely playing a role in the loss of hair in the human lineage [66–68]. In contrast, we found that keratin 6 and keratin 1 are upregulated in psoriasis; both are involved in maintaining skin integrity and inflammation response, likely playing crucial mechanistic roles in plaque skin formation and the psoriatic process. *KRT77* was observed to be significantly downregulated in psoriatic plaques (*P*_adj_ < 0.05); it has been shown to be primarily expressed within an eccrine gland context [69]. Because psoriatic tissue has little sweat activity even under sweat-inducing stimuli, *KRT77* is a primary candidate to investigate further in the context of sweating, as its downregulation may be related to the reduction of sweat response in psoriatic plaques.

### Dynamic regulation of protease activity underlies human skin evolution and psoriasis

We found that a cluster of 15 kallikrein protease genes on Chromosome 19 and a cluster of 9 serpin protease inhibitor genes on Chromosome 18 are differentially regulated in both human evolution and psoriatic plaque formation (Supplementary Fig. S5A–C). Kallikrein proteases play an essential role in skin barrier function and are implicated in skin shedding and psoriasis [70]. Similarly, serpins are major contributors to skin function, and their activity is connected to several autoimmune skin conditions [71]. The functions of kallikrein proteases and serine protease inhibitors are intertwined in the regulation of epidermal homeostasis, so it is unsurprising that multiple *KLK* and serpin genes are upregulated in psoriatic plaques [70]. *SERPINB3* and *SERPINB4* are significantly upregulated in psoriatic plaques and are relatively recent duplicates of each other (Supplementary Fig. S5); their expression affects expression of *S100A8* in a murine model and may play a role in the initiation of a pro-inflammatory cascade in skin [71]. In parallel, *SERPINB11* and *SERPINB13* are human-specifically downregulated, while kallikrein 14, a major player in wound healing and inflammatory skin disorders, is upregulated more than 100 fold [70]. Additionally, *KLK6* is significantly downregulated in the human lineage but upregulated in psoriatic tissue. This gene is particularly noteworthy because it is involved in psoriatic arthritis, linking skin inflammation with another systemic autoimmune response [70, 72, 73]. The complex evolution of serpins and kallikreins in the human lineage and differential regulation of these genes in psoriasis hints at a dynamic interplay through which skin homeostasis is maintained and stress response is realized.

## CONCLUSIONS

We first analyzed gene expression in a novel psoriatic RNA sequencing dataset, adding to previous research on psoriasis that shows a general enrichment of genes involved in immune response and cellular adhesion. We then investigated gene expression in psoriasis compared to humans and non-human primates without psoriasis to identify human-specific trends linked to skin evolution and immunity. We found 67 human-specific genes mainly involved in skin barrier function and development located in 6 clusters across the human genome. We highlight clustering of histones, protocadherins, keratins, serpins, kallikreins and the EDC, which play roles in human skin function and hint to recent evolution of human skin homeostasis with the environment. We argue that the dynamic interplay between gene expression in skin, human evolution and environmental adaptation is important to how skin-environment homeostasis and perception are achieved, specifically in psoriasis.

The skin co-evolves with the environment to protect from and perceive external stimuli and is a crucial part of the larger bodily system. Human-specific gene expression in skin, specifically related to sensation and barrier function, is regulated differently in individuals with psoriasis, likely due to increased cutaneous innervation in psoriatic plaques. The increased number of nerve endings in psoriatic plaques cause increased sensation, exacerbating itchiness and pain from the lesions themselves [60]. In fact, nerve injury and denervation have been shown to decrease psoriatic plaque development [20, 60, 74]. Increased innervation can lead to increased perception of the external environment causing flooding of information that disrupts bodily homeostasis and affecting a feedback loop where systemic psoriatic symptoms are exacerbated by external stressors [5, 12, 19–21]. It is possible that the increase in nerve endings in psoriatic plaques aid in stress response and wound healing. However, increased and ongoing sensitivity and perception of external stimuli can also lead to chronic autoimmune expression, increased rates of neuroinflammatory pathway activation in skin tissues, and hyper-perception of external/perceived stressors [59, 75]. In this context, it is tempting to link the collective upregulation in expression of virtually all transcripts of clustered protocadherins in human skin with cutaneous sensory neuron activity [60]. The downregulation of this cluster in psoriatic lesions may be part of the complex involvement of sensory nerves in the immune response associated with psoriasis [67, 68]. Future studies of protocadherins at the single-cell level provide an exciting venue to understand neuroimmune pathways, shed light on putative differences in perception between humans and non-human primate relatives, and explain remissions of psoriasis following nerve damage or denervation [60, 67, 69, 70].

Psoriasis is a complex autoimmune response comprising inflammation across the body, primarily diagnosed by lesions on the skin. People with psoriasis have high rates of comorbidities with other complex autoimmune and metabolic conditions as well as cardiovascular disease and mental illness. These conditions that are shaped by both genetic as well as environmental factors, making the feedback loop between the environment, the skin and the neuroimmune system an ideal place to study evolution of the skin transcriptome [12, 13]. Given the psychoneuroimmunological relationship between allostatic load and inflammation of tissue, as well as the evolved relationship between the skin and the external environment, it is important that we not only look at the symptoms of stress in skin, but also its root causes. While prior work has often separated the genetic and/or evolutionary basis of psoriasis from the role of the environment and evolved skin/bodily homeostasis, this method often ignores the interconnectedness of the past and present within a larger global picture of health.

Many discussions of human genome variation and associated biomedical implications ignore the impacts of ongoing direct and accumulated environmental impact, though this is thankfully changing. We envision two major directions for future research. First, it will be important to elucidate mechanisms through which evolutionary changes in the genome and transcriptome translate into immune variation. Second, an integrative understanding of human skin evolution and immune-mediated processes must address epigenetic expression of chronic stress in the skin, as well as the systemic and comorbid nature of autoimmune conditions.

## IMPLICATIONS

It is our hope that this article contributes a framework and analysis of transcriptomic data that furthers understanding of human skin-environment co-evolution and skin perception of the environment in psoriasis. This study can provide more evolutionary context for medical practitioners and public health professionals in understanding broader systemic, environmentally situated ways to develop preventative measures and treatment options for psoriasis and other autoimmune issues.

## Supplementary data

Supplementary data are available at *EMPH* online.

## Data availability

The processed RNAseq data for both psoriatic skin and primate skin samples are available in the supplementary tables. The raw sequence data for the primate RNAseq was previously published [33] and is available through DDBJ Sequence Read Archives (accessions DRX121122–DRX121135). The raw sequence data for the lesional and non-lesional skin from psoriatic patients have been uploaded to GEO (accession GSE183820).

**Conflict of interest**: None declared.

## Supplementary Material

eoab042_Supplementary_Data
