# Supplementary material for: Evolutionary context of psoriatic immune skin response
Source: Evol Med Public Health. 2021 Dec 1;9(1):474–86. doi: 10.1093/emph/eoab042 (PMC8830311; doi:10.1093/emph/eoab042)
Supplement: eoab042_Supplementary_Data [file eoab042_Supplementary_Data.zip › HRP-502 -Consent_June2020.pdf]

Permission to Take Part in a Human Research Study

University at Buffalo Institutional Review Board (UBIRB)
Office of Research Compliance | Clinical and Translational Research Center Room 5018
875 Ellicott St. | Buffalo, NY 14203
UB Federalwide Assurance ID#: FWA00008824

Adult Consent to Participate in a Research Study

Title of research study: Evolutionary and clinical examination of genetic, microbial and immune response variation in epidermal differentiation complex to understand common immune-mediated skin diseases

Version Date: *May 4, 2017*

Investigator: *Omer Gokcumen, Ph.D. and Animesh A. Sinha, MD, PhD*

Key Information: The following is a short summary of this study to help you decide whether or not to be a part of this study. More detailed information is listed later on in this form.

Why am I being invited to take part in a research study?

You are being asked to participate in a research study investigating the evolutionary and genetic causes of immune-mediated skin diseases. Either as a patient for such as disease or as a healthy control, your participation will enable us to specifically ask whether certain genetic variants affect susceptibility to these diseases through affecting skin microbiome. The purpose of this document is to provide you with information to consider in deciding whether to participate in this research study. Your consent should be made based on your understanding of the nature and risks of the procedure. Please ask questions if there is anything you do not understand. Your participation is voluntary and will have no effect on the quality of your medical care if you choose not to participate.

What should I know about a research study?

- Someone will explain this research study to you.
- Whether or not you take part is up to you.
- You can choose not to take part.
- You can agree to take part and later change your mind.
- Your decision will not be held against you.
- You can ask all the questions you want before you decide.

Why is this research being done?

The purpose of the research is to investigate causes of several skin diseases, including atopic dermatitis, psoriasis, and pemphigus vulgaris. Specifically, we ask whether inherited variation in our genetic code change the differences microbes living on our skin and how our body reacts to these microbes. This in turn allow us to find connected "packages" of genetic, microbial and immune factors that lead to immune-mediated skin diseases. We base this work to our recent published studies where we asked why, from an evolutionary point of view, variation in the genomes that predispose to skin disorders remain in human populations. We will also investigate the differences gene expression patterns between the biopsies collected from psoriatic and the healthy skin. This analysis will allow us to understand the specific genes that are involved in psoriasis. The information gained in this study

will advance our understanding of the inherited and environmental mechanisms that lead to immune-mediate skin disorders and can be expected to reveal novel molecular targets for therapy.

How long will the research last and what will I need to do?

On your first day of study participation, it will take approximately 30 to 45 minutes for you to sign the consent, fill out an intake questionnaire and donate samples. The samples collected will be used immediately or frozen for use at a later time point. We will keep your data in a study database and you may be asked for 1-3 follow-up visit(s) at later time point(s), up to a few years from now. These subsequent visits are rarely necessary and is completely voluntary. They will take a much shorter amount of time (>10 minutes).

More detailed information about the study procedures can be found under ***"What happens if I say yes, I want to be in this research?"***

Is there any way being in this study could be bad for me?

While rare, potential risks of blood draw and skin biopsy procedures are slight pain and bleeding and/or bruising at the blood drawing or biopsy site, as well as temporary faintness. The results from the analyses of your samples will be used only for science. No names will ever be used and data will be kept confidential. More detailed information about the risks of this study can be found under ***"Is there any way being in this study could be bad for me? (Detailed Risks)"***

Will being in this study help me in any way?

There is no direct benefit to you for your participation in this study. However, your willingness to take part may help doctors better understand and/or treat others who have several skin diseases, and the knowledge gained may eventually be advantageous to all patients affected with a variety of immune-mediated disorders.

What happens if I do not want to be in this research?

Your participation in this research study is voluntary. You may choose not to enroll in this study.

Detailed Information: The following is more detailed information about this study in addition to the information listed above.

Who can I talk to?

If you have questions, concerns, or complaints, or think the research has hurt you, talk to the research team at at the University at Buffalo (contact: Dr. Kristina Seiffert-Sinha, MD; Title: Research Assistant Professor; Department of Dermatology, School of Human Medicine and Biomedical Sciences, Phone Number: 716-842-2218). You may also contact the research participant advocate at 716-888-4845 or researchadvocate@buffalo.edu.

This research has been reviewed and approved by an Institutional Review Board ("IRB"). You may talk to them at (716) 888-4888 or email ub-irb@buffalo.edu

if:

- You have questions about your rights as a participant in this research
    - Your questions, concerns, or complaints are not being answered by the research team.
    - You cannot reach the research team.
    - You want to talk to someone besides the research team.

- You want to get information or provide input about this research.

You may also contact the research participant advocate at 716-888-4845 or researchadvocate@buffalo.edu.

This research has been reviewed and approved by an Institutional Review Board ("IRB"). An IRB is a committee that provides ethical and regulatory oversight of research that involves human subjects. You may talk to them at (716) 888-4888 or email ub-irb@buffalo.edu if:

- You have questions about your rights as a participant in this research
- Your questions, concerns, or complaints are not being answered by the research team.
- You cannot reach the research team.
- You want to talk to someone besides the research team.
- You want to get information or provide input about this research.

How many people will be studied?

We expect to enroll approximately up to 200 people in this research study. People will primarily be enrolled at the outpatient clinics of the Department of Dermatology at the University at Buffalo.

What happens if I say yes, I want to be in this research?

If you decide to take part in this research study, you will undergo the following procedures: you will be asked intake questions regarding your age and ancestral background. In addition, if you have one of the target diseases (atopic dermatitis, psoriasis, or pemphigus vulgaris) we will collect information regarding diagnosis, manifestation and course of disease as well as your medical history and all medications you are currently receiving. Then, we will sample the following:

- 2 vials (~ 1 Tablespoon) of blood will be taken for DNA and gene expression analyses both from patients and from healthy control subjects. This analysis provides a general look at which of your genes are active in a given time and help us understand the functioning of your immune system.

- 6 skin swab samples (3 from skin lesions and 3 from healthy skin) will be taken for microbiome analysis. If you are designated as a healthy control, we will get 3 samples only from the healthy skin. This analysis will allow us to examine what kind of microbes live on healthy skin and on skin lesions.

- 2 skin punch biopsy samples (1 from lesion and 1 from healthy tissue) for will be taken for comparative expression analyses. If you are designated as a healthy control, we will get 1 sample only from the healthy tissue. Skin biopsy is the primary technique for obtaining diagnostic full-thickness skin samples and it involves a a circular blade that is rotated down through 3- to 4-mm of skin an get a cylindrical core of skin sample. This sample will allow us to look at the skin-specific activity of thousands of genes to understand the cellular responses to different disease states as well as presence of certain microbes. The personnel who collect the biopsies will provide the necessary cleaning and treatment for the biopsy site. There will be no special treatments necessary other than keeping the site clean. If any complications occur, you can contact the Dr. Kristina Seiffert at Phone Number: 716-842-2218)

It will take approximately 30 to 45 minutes to sign the consent, fill out the intake and donate samples. You will only interact with the study personnel listed on this consent or designees of the Department of Dermatology at the University at Buffalo, Buffalo, NY. After these procedures, you will be monitored for extra 10 minutes before release.

Permission to Take Part in a Human Research Study

If there are any questions regarding your disease status or other relevant information, we may follow up with you by mail, email or phone. You may also be asked for a follow-up visit at a later time point.

The research will be carried out at

- Outpatient clinics of the Department of Dermatology at the University at Buffalo.
- The Dermatology Laboratory at the Clinical and Translational Research Center in Buffalo.
- Department of Biological Sciences at the University at Buffalo.

All procedures are being performed as part of a research study and not as part of your standard medical care.

The samples will be used immediately or stored frozen for use at a later time-point. Unused samples and associated data will be kept for a minimum of 3 years after completion of the study and will subsequently be destroyed.

What happens if I say yes, but I change my mind later?

You can leave the research at any time it will not be held against you.

Is there any way being in this study could be bad for me? (Detailed Risks)

There are no known risks for sampling by skin swabs.

While rare, potential risks of blood draw and skin biopsy procedures are slight pain and bleeding and/or bruising at the blood drawing or biopsy site, as well as temporary faintness. There is a small risk that the blood drawing site could become infected. Potential risks of skin biopsy are slight pain and bleeding and/or at the biopsy. There is a small risk that the skin biopsy site could become infected. The biopsy site may heal with a small scar.

Information related to you will be treated in strict confidence to the extent provided by law. Your identity will be coded and will not be associated with any published results. Your code number and identity will be kept in a locked file of the Principal Investigator. However, there is a risk of loss of confidentiality should our safeguards be breached.

The results from the analyses of your samples will be used only for science. No names will ever be used and data will be kept confidential. You will not be told of these possible tests, nor will you receive results of any of these tests.

What happens to the information collected for the research?

Efforts will be made to limit the use and disclosure of your personal information, including research study and medical or education records, to people who have a need to review this information. We cannot promise complete secrecy. Organizations that may inspect and copy your information include the IRB and other representatives of this organization. A small portion of the samples will be stored for future testing. The nature of this testing is not known at this time; however, investigators may look at inherited factors which are related to diseases by examining DNA obtained from the stored samples. This DNA may be used within the context of this project, for analysis of genetic variation that may regulate hormones, growth factors and other processes that may influence skin diseases. After removal of identifiable private information, the biospecimens could be used for future research studies or distributed to another investigator for future research studies without additional informed consent from the subject or the LAR. The samples will remain the property of the Department of Dermatology at the

University at Buffalo. The blood samples and the DNA obtained from the samples are stored and tested with an identifying number, and your name will not appear on the stored samples.

Federal law provides additional protections of your medical records and related health information. These are described in the HIPAA section of this document.

Can I be removed from the research without my OK?

The principal investigator of the study can remove you from the research study without your approval. Possible reasons for removal include the development of a condition with a known effect on the immune system (other than autoimmune conditions).

What else do I need to know?

If you need medical care during taking part in this research study, contact the investigator and medical care will be made available. Generally, this care will be billed to you, your insurance or other third party.

Who is paying for this research?

This research is currently funded by Genome, Environment and Microbiome initiative of the University at Buffalo.

Will I get paid for my participation in this research?

You will not be paid for taking part in the study. However, you will be reimbursed $25.00 for your time and travel for one blood draw and skin swab visit, and $50 for the skin biopsy visit (this includes the follow up visit for stitches removal in approximately 2 weeks after the procedure).

You will not receive any payments at your study visits. It may take up to 6 weeks to process and receive each payment. If you do not complete the entire study, you will be reimbursed only for the visits that were completed while you were in the study.

Payment for taking part in a research study may be considered taxable income. If this payment is more than $600.00 in any one calendar year, the study doctor or clinic will have to report this to the Internal Revenue Service (IRS). This will be reported using a 1099 (Miscellaneous Income) form. This form will be issued to you and a copy will be sent to the IRS.

What will happen to my information and samples?

The data points obtained from individual samples are meaningless to the individual patient unless they are compared to and viewed in the context of samples obtained from a large number of subjects. Our findings will be published in scientific journals and are freely accessible or can be obtained from the investigators. You will not be notified of your individual findings as part of this research study unless you specifically request certain analyses.

Permission to Take Part in a Human Research Study

If you like to withdraw from this study you may do so by letting us know in writing (e-mail or letter). After withdrawal, you may choose to have these samples destroyed. If the samples are already analyzed, the data will remain in the study anonymously.

HIPAA: Authorization for the Use and Disclosure of Identifiable Health Information for Research Purposes

This section describes information about you and about your health that will be obtained by the researchers when you participate in the research study. Health information is considered "protected health information" when it may directly identify you as an individual. By signing this form you are agreeing to permit the researchers and/or other parties (described in detail below) to have access to this information. If there are any parts of this form that you do not understand, please be sure to ask us for further clarification.

A. What protected health information will be collected about you as part of this research study?

__√__ Information from your full medical records. Specifically, we are interested in the progression of the skin-related symptoms.

__√__ New Health Information created from study related tests, procedures, visits, and/or questionnaires as described in this consent form.

B. Who is authorized to provide or collect this information?

_____ KALEIDA Health, Buffalo NY
__√_ Principal Investigator or designee
_____ ECMC Healthcare Network, Buffalo NY
_____ University at Buffalo School of Dental Medicine
_____ Other (identify):____

C. With whom may your protected health information be shared?

Your health information may be shared with others outside of the research group for purposes directly related to the conduct of this research study or as required by law, including but not limited to:

Your information may also be shared with individuals or entities responsible for general administration, oversight and compliance of research activities. Examples of this include the institution's Privacy and Security Officers or other internal oversight staff, Safety Monitoring Boards, an Institutional Review Board, The Research Foundation of the State University of New York, University at Buffalo Foundation Services, and accrediting bodies, or with certain government oversight agencies that have authority over the research including the Department of Health and Human Services (HHS), the Food and Drug Administration (FDA), the National Institutes of Health (NIH), and the Office of Human Research Protections (OHRP). Your information may also be shared with other entities as permitted or required by law. All reasonable efforts will be used to protect the confidentiality of your individually identifiable health information that may be shared with others as described above.

All reasonable efforts will be used to protect the confidentiality of your protected health information. There is the potential for individually identifiable information and the associated health information obtained with this authorization to be re-disclosed by the recipient(s). After such a disclosure, the information may no longer be protected by the terms of this authorization against further re-disclosure.

D. How long will this information be kept by the Principal Investigator?

___√__ b. This authorization will expire at the end of the research study. After that time, this authorization may not be used to acquire additional information about you.

E. What are your rights after signing this authorization?

You have the right to revoke this authorization at any time. If you withdraw your authorization, no additional efforts to collect individually identifiable health information about you will be made. You should know, however, that protected health information acquired using this authorization prior to its withdrawal may continue to be used to the extent that the investigator(s) have already relied on your permission to conduct the research. If you chose to withdraw this authorization, you must do so in writing to the following individual(s):

Kristina Seiffert-Sinha, MD, Research Assistant Professor, Department of Dermatology, Jacobs School of Medicine and Biomedical Sciences, 875 Ellicott Street, CTRC Room 6085, Buffalo, NY 14203; email: krs2002@buffalo.edu.

The organization(s) responsible for administering this research – University at Buffalo, Department of Dermatology.

If you send us a request to withdraw your authorization, we will forward that request to the institutions we have shared it with in order to collect your individually identifiable health information.

F. What will happen if you decide not to sign this authorization?

Refusing to sign this authorization will not affect the present or future care you receive at this institution and will not cause any penalty or loss of benefits to which you are otherwise entitled. If you decide not to sign this authorization, you will not be able to participate in the research study.

Signature Block for Capable Adult

Your signature documents your permission to take part in this research. By signing this form you are not waiving any of your legal rights, including the right to seek compensation for injury related to negligence or misconduct of those involved in the research.

Permission to Take Part in a Human Research Study

Signature of subject	Date
Printed name of subject	
Signature of person obtaining consent	Date
Printed name of person obtaining consent	
