# Supplementary material for: Evolutionary context of psoriatic immune skin response
Source: Evol Med Public Health. 2021 Dec 1;9(1):474–86. doi: 10.1093/emph/eoab042 (PMC8830311; doi:10.1093/emph/eoab042)

SUPPLEMENTARY DOCUMENTATION

Table S1: Patient Skin Punch Sample Information

	**Sex**	**Ethnicity**	**Age (years)**	**PASI score**	**Biopsy site lesional**	**Biopsy site nonlesional**
**Pso2**	Male	European	34	8.1	lower back	lower back
**Pso3**	Female	European	53	3.8	upper arm	upper arm
**Pso5**	Male	European	68	1	elbow	elbow

Table S2: Psoriasis and Primate Sample Information

**project**	**sample**	**individual**	**condition**	**mammal**
OG_pso	Pso2A	Pso2	plaque	human
OG_pso	Pso2B	Pso2	control	human
OG_pso	Pso3A	Pso3	plaque	human
OG_pso	Pso3B	Pso3	control	human
OG_pso	Pso5A	Pso5	plaque	human
OG_pso	Pso5B	Pso5	control	human
ST_prim	chimp2	chimp2	primate	chimp
ST_prim	chimp7	chimp7	primate	chimp
ST_prim	chimp10	chimp10	primate	chimp
ST_prim	gorilla9	gorilla9	primate	gorilla
ST_prim	gorillaFCC	gorillaFCC	primate	gorilla
ST_prim	human4	human4	primate	human
ST_prim	human5	human5	primate	human
ST_prim	human6	human6	primate	human
ST_prim	humanOba2	humanOba2	primate	human
ST_prim	humanOji	humanOji	primate	human
ST_prim	oran3	oran3	primate	orangutan
ST_prim	oran8	oran8	primate	orangutan
ST_prim	oranCHK	oranCHK	primate	orangutan

Table S3: Gene expression in psoriatic skin
Tab 1: GROUPED Comparision between lesional and nonlesional skin expression in psoriasis
Tab 2: Normalized read-depth for individual samples
Tab 3: PAIRWISE Comparision between lesional and nonlesional skin expression in psoriasis

Table S4: Go analysis enrichment
Tab 1: Genes upregulated in psoriasis lesional-nonlesional analysis
Tab 2: Genes downregulated in psoriasis lesional-nonlesional analysis
Tab 3: Genes upregulated in human-chimpanzee analysis
Tab 4: Genes downregulated in human-chimpanzee analysis

Table S5: Gene expression in human and nonhuman primate skin
Tab 1: Comparison between human and chimpanzee skin expression
Tab 2: Normalized read-depth for individual samples

Table S6: Subset of differential expression in human lineage and psoriasis
Tab 1: Differentially expressed genes in the human lineage and psoriatic lesions
Tab 2: Differentially expressed genes by cluster

Figure S1. Comparison of gene expression in psoriasis transcriptome. A. Plot showing matches among top 100 differentially expressed genes (ranked based on p-values) from four psoriasis RNAseq datasets. **B.** Venn diagram of overlaps in top 100 differentially expressed genes from datasets in A.

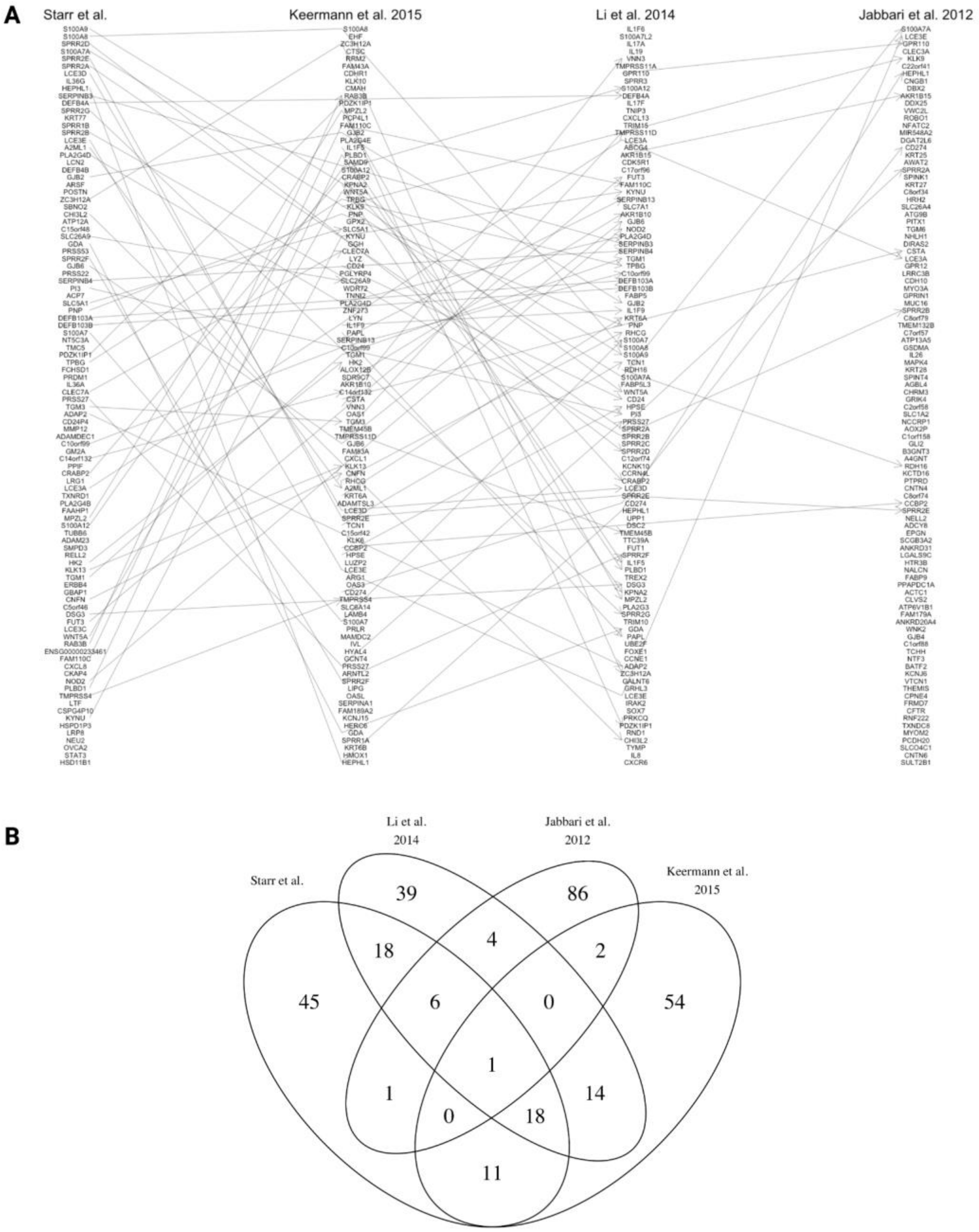

Figure S2. Comparison of log10 average normalized counts from chimpanzee transcriptome reads mapped to human (Hg38, x-axis) and chimpanzee (PanTro3, y-axis) reference genomes.

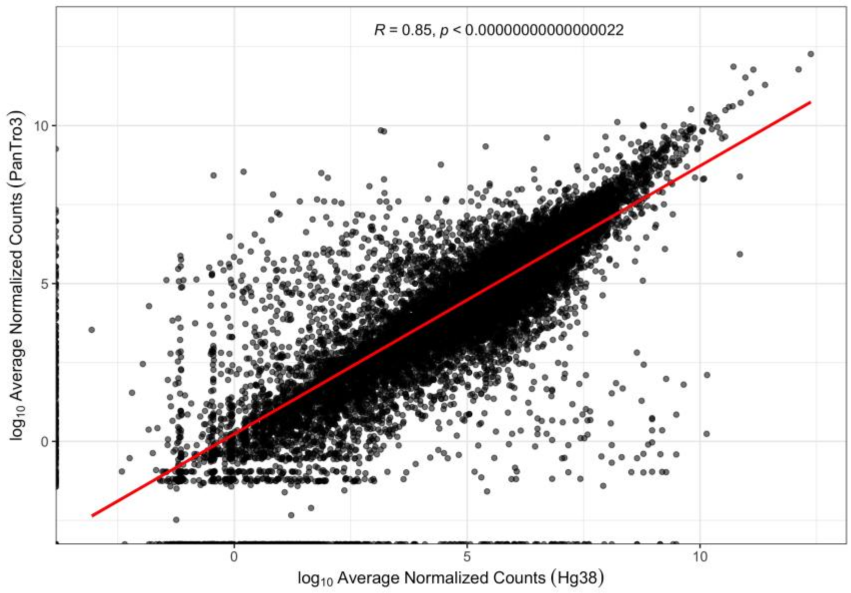

Figure S3. UCSC Genome Browser (hg38) screenshots of genomic clusters highlighted in the main text. The genomic locations, gene annotations, and segmental duplications are shown. Exonic duplications are indicated by red arrows and manually annotated. **A.** Epidermal differentiation complex (chromosome 1). **B.** Protocadherins (chromosome 5). **C.** Histones (chromosome 6). **D.** Keratins (chromosome 12). **E.** Serpins (chromosome 18). **F.** Kallikreins (chromosome 19).

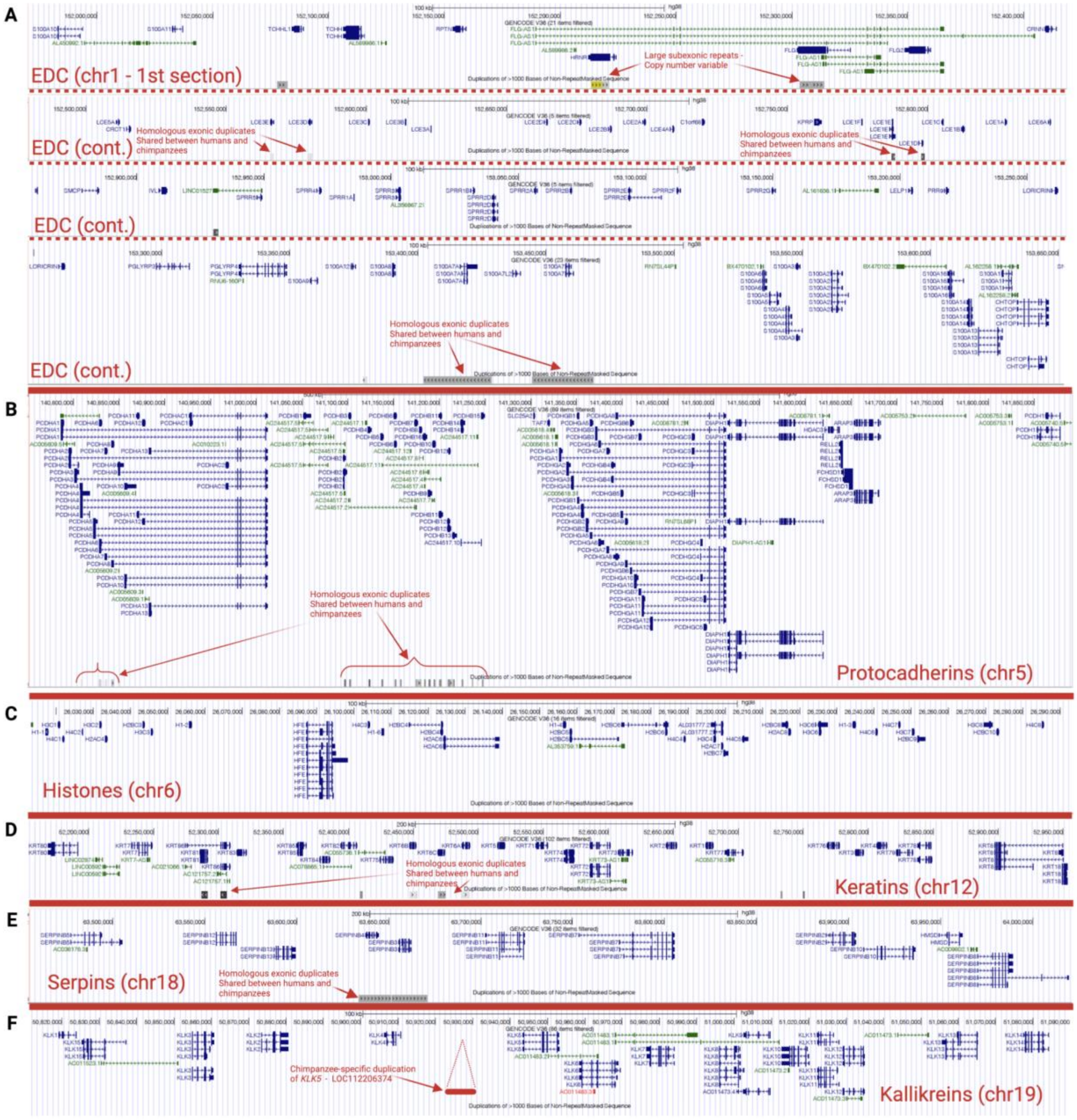

Figure S4. IGV screenshot of RNAseq sequence reads mapped to Hg38. We chose this region because it harbors duplicated genes, SERPINB3 and SERPINB4, which show significant expression differences between our samples. The top two rows are data from Arakawa et al. and the bottom two rows are data produced from this study. The mapping is done with similar parameters for all 4 samples (see **Methods**). The scales of expression are adjusted for each sample and are schematically shown on the right barplot; blue indicates data from Arakawa et al. and orange indicates data from this study. The histograms show read depth at each location; gray indicates optimal matches and multicolored lines indicate mismatches. Even though *SERPINB3* and *SERPINB4* are relatively recent duplicates shared by humans and chimpanzees, the RNAseq reads can distinguish between these two copies and in all instances *SERPINB4* has higher expression. Note that we found no significant differences between humans and chimpanzees for these genes, but clear expression differences between psoriatic and nonpsoriatic skin in humans.

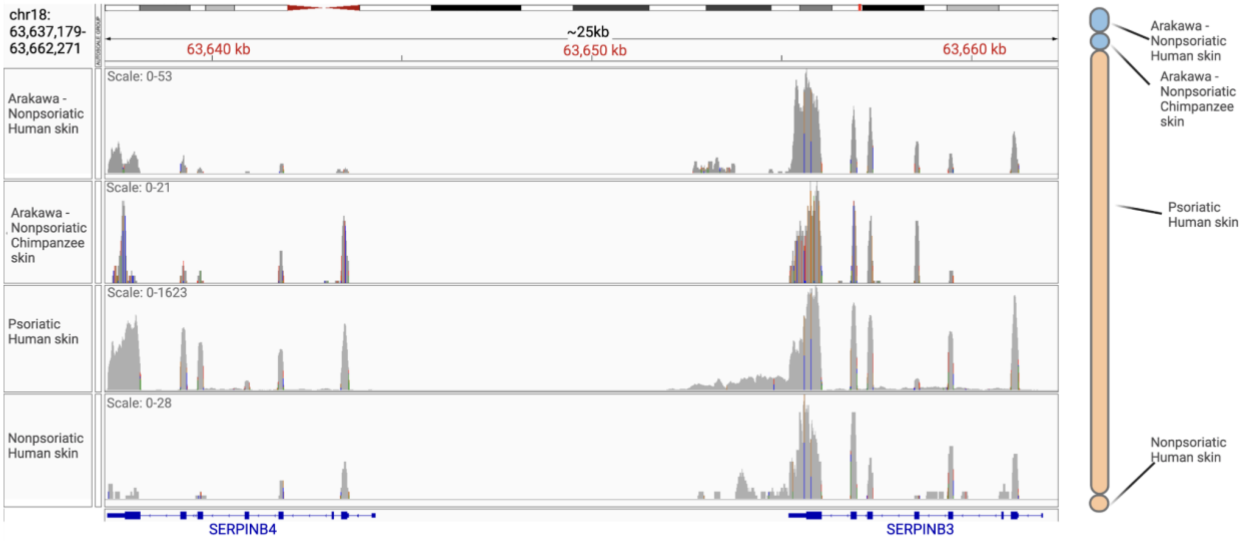

Figure S5. Gene expression in keratin, serpin, and kallikrein clusters. The barplots show the log2 fold changes of gene expression in the human lineage **(A)** and in psoriasis **(B)** in a keratin gene cluster on chromosome 12, serpin cluster on chromosome 18, and kallikrein cluster on chromosome 19. **C.** The chromosomal location of genes, where those with significant human-lineage and psoriatic-expression differences match panels A and B.

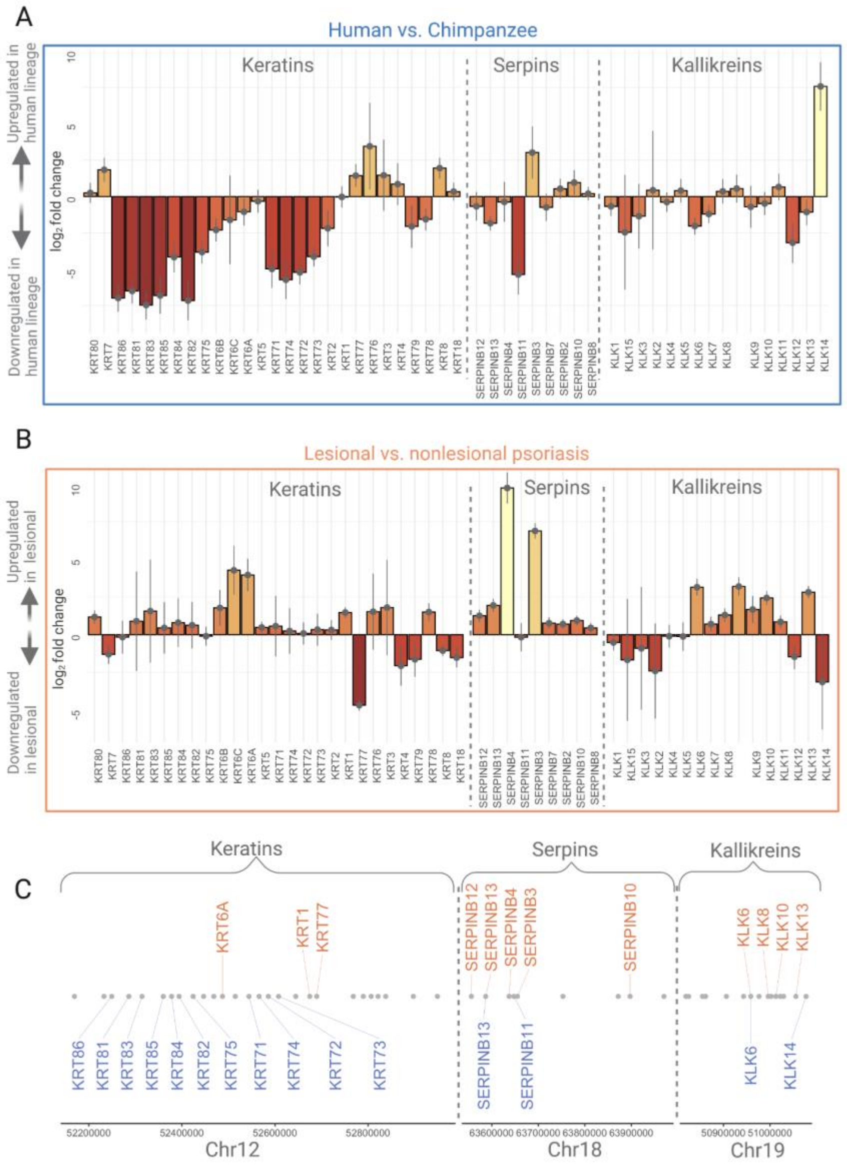

Supplement: eoab042_Supplementary_Data [file eoab042_Supplementary_Data.zip › Supplementary_EDITED.pdf]
